# Supplementary material for: Sex-specific cardiac remodeling in early and advanced stages of hypertrophic cardiomyopathy
Source: PLoS One. 2020 May 5;15(5):e0232427. doi: 10.1371/journal.pone.0232427 (PMC7199944; doi:10.1371/journal.pone.0232427)
Supplement: S1 Table — Abbreviations: F (female); M (male). (DOCX) [file pone.0232427.s001.docx]

**Supplementary Table 1. Controls**

| Number | sample code | sex (F/M) | Age (years) |
| --- | --- | --- | --- |
| 1 | 3073 | F | 41 |
| 2 | 3108 | F | 37 |
| 3 | 3112 | F | 23 |
| 4 | 3141 | M | 52 |
| 5 | 3145 | M | 39 |
| 6 | 3149 | M | 56 |
| 7 | 3160 | M | 21 |
| 8 | 3162 | F | 37 |
| 9 | 3164 | M | 61 |
| 10 | 3168 | F | 19 |
| 11 | 4008 | F | - |
| 12 | 4013 | M | 23 |
| 13 | 4015 | M | 19 |
| 14 | 4021 | F | 53 |
| 15 | 4043 | M | 14 |
| 16 | 4049 | M | 65 |
| 17 | 4062 | M | 55 |
| 18 | 4104 | F | 49 |
| 19 | 5003 | M | 37 |
| 20 | 5084 | F | 49 |
| 21 | 5086 | M | 29 |
| 22 | 5128 | M | - |
| 23 | 6008 | M | 40 |
| 24 | 6020 | F | 38 |
| 25 | 6034 | F | 55 |
| 26 | 6056 | F | 42 |
| 27 | 7040 | M | 37 |
| 28 | 7044 | M | 63 |
| 29 | 7054 | M | - |
| 30 | 8004 | F | - |

Abbreviations: F (female); M (male).
